# Adaptive Evolution of the Venom-Targeted vWF Protein in Opossums that Eat Pitvipers

**DOI:** 10.1371/journal.pone.0020997

**Published:** 2011-06-22

**Authors:** Sharon A. Jansa, Robert S. Voss

**Affiliations:** 1 Department of Ecology, Evolution and Behavior, and J.F. Bell Museum of Natural History, University of Minnesota, St. Paul, Minnesota, United States of America; 2 Department of Mammalogy, American Museum of Natural History, New York, New York, United States of America; Instituto Butantan, Brazil

## Abstract

The rapid evolution of venom toxin genes is often explained as the result of a biochemical arms race between venomous animals and their prey. However, it is not clear that an arms race analogy is appropriate in this context because there is no published evidence for rapid evolution in genes that might confer toxin resistance among routinely envenomed species. Here we report such evidence from an unusual predator-prey relationship between opossums (Marsupialia: Didelphidae) and pitvipers (Serpentes: Crotalinae). In particular, we found high ratios of replacement to silent substitutions in the gene encoding von Willebrand Factor (vWF), a venom-targeted hemostatic blood protein, in a clade of opossums known to eat pitvipers and to be resistant to their hemorrhagic venom. Observed amino-acid substitutions in venom-resistant opossums include changes in net charge and hydrophobicity that are hypothesized to weaken the bond between vWF and one of its toxic snake-venom ligands, the C-type lectin-like protein botrocetin. Our results provide the first example of rapid adaptive evolution in any venom-targeted molecule, and they support the notion that an evolutionary arms race might be driving the rapid evolution of snake venoms. However, in the arms race implied by our results, venomous snakes are prey, and their venom has a correspondingly defensive function in addition to its usual trophic role.

## Introduction

Animal venoms are complex mixtures of toxic proteins and peptides that induce a wide variety of destructive physiological effects. Recent studies of snake, scorpion, and gastropod venoms provide compelling evidence for the rapid evolution of genes encoding many toxic proteins [1]. For example, venom toxin genes often belong to large multi-gene families with rapidly evolving protein-coding regions that exhibit high ratios of replacement to silent substitutions [2]–[10]. Although evolutionary explanations for these and other unusual properties of animal-venom genes commonly invoke the metaphor of an “arms race” between venomous animals and their prey [5], [7], [8], [11], the appropriateness of this metaphor remains to be demonstrated. Whereas an arms race implies reciprocal adaptations and counter-adaptations in a coevolutionary contest for which no stable equilibrium exists [12], there appears to be no published evidence for rapid adaptive evolution of molecular traits that might confer toxin resistance in routinely envenomed taxa. Here we report such evidence from an unusual predator-prey relationship between pitvipers (members of the viperid snake subfamily Crotalinae) and opossums (members of the marsupial family Didelphidae).

Pitvipers are ambush predators that detect the elevated body temperatures of endothermic prey—birds and mammals—with an infrared-sensitive pit organ located between the eye and nostril [13]. Like other venomous snakes, pitvipers subdue their prey with a potent blend of toxic molecules secreted by specialized cephalic glands [14]–[16]. Pitviper venom, powerfully hemorrhagic in most species, is delivered to the bloodstream of the victim through hollow, needlelike fangs that are embedded hypodermically in a lightning-fast stabbing bite [17]. Small mammals bitten by pitvipers usually die quickly of cardiovascular shock induced by the synergistic action of many different venom components [18]. The latter commonly include A_2_ phospholipases, zinc-dependent metalloproteinases, C-type lectin-like proteins, serine proteases, and disintegrins [11], [19], [20].

**Figure 1:**
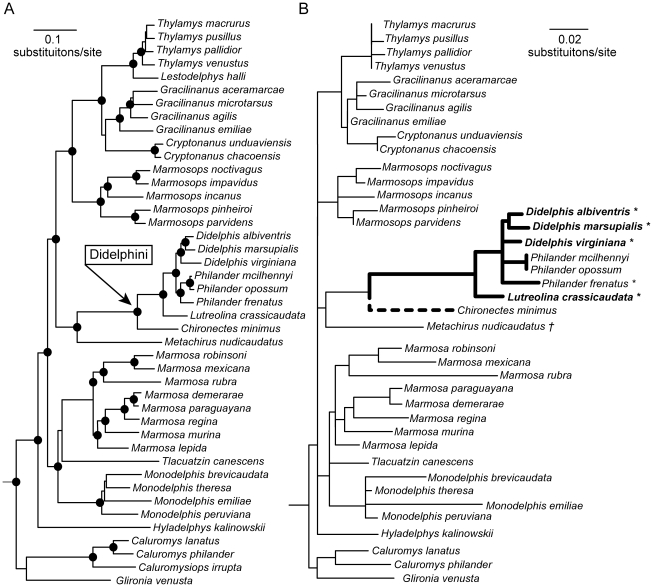
Phylogenetic trees of opossums. **A**. The phylogeny of didelphids resulting from a mixed-model Bayesian analysis of a combined-data matrix comprising DNA sequences from five nuclear protein-coding genes and morphological data [28]. Nodes that received Bayesian posterior probability values ≥0.95 in this analysis are indicated with black circles. **B**. The topology from **A** excluding *Lestodelphys* and *Caluromysiops*, (for which no vWF sequences are available). Branch lengths are shown as the estimated number of amino acid substitutions in vWF, assuming the JTT model of amino acid substitution as implemented in PAML [74]. Taxa that are known to eat pitvipers are indicated in bold; those that are known to exhibit resistance to pitviper venom are indicated with an asterisk. *Metachirus* (indicated with a dagger) has been challenged with pitviper venom but does not exhibit resistance. Branches that were included in the foreground for branch-site tests are shown with solid heavy lines. Venom resistance of *Chironectes* is unknown; therefore, this taxon was included in one set of branch-site tests and excluded from the other (indicated with a dashed heavy line). For the purpose of this analysis, *Didelphis marsupialis* includes its dubiously distinct sister taxon *D. aurita*.

Despite such formidable biochemical weaponry, some opossums eat pitvipers with impunity. This extraordinary behavior was first reported by the Spanish naturalist Félix de Azara [21] for the lutrine opossum (*Lutreolina*), and it has subsequently been documented for several species of common opossums (*Didelphis*). Opossums that prey on pitvipers appear to exhibit no behavioral precautions while subduing these dangerous snakes, and they are often bitten in the process [22], [23]. Rather, their impunity derives from endogenous venom resistance, a phenomenon that has been convincingly demonstrated by numerous *in vivo* and *in vitro* assays using *Didelphis*, *Lutreolina*, and the gray four-eyed opossum *Philander* [24]–[27]. As far as known, all didelphids that are known to eat pitvipers and/or to be venom resistant belong to the tribe Didelphini [28] (Fig. 1). It is also known that the brown four-eyed opossum (*Metachirus*), the sister taxon to Didelphini, is not venom resistant [29].

In an early publication on venom resistance in didelphids, Kilmon [24] proposed two explanations for this phenomenon: either (1) the molecular targets for venom toxins are absent, or (2) something in the tissue inactivates venom toxins before they reach their targets. The first alternative is implausible because snake venoms disrupt basic biological processes by targeting physiologically indispensible molecules (e.g., those involved in hemostasis [19], [20]). Research on venom resistance in mammals has therefore focused almost exclusively on the discovery of toxin-neutralizing serum factors, most of which are enzyme inhibitors [30]–[34]. However, many snake venom toxins are not enzymes [11], so additional mechanisms of venom resistance may be necessary to explain the complete immunity to pitviper envenomation of some opossums. Among other nonenzymatic components of pitviper venom are C-type lectin-like proteins (CLPs), a functionally diverse family of ligand-binding toxins that disrupt hemostasis by targeting a wide range of plasma proteins and blood cell types [6], [35]–[37]. Because CLPs are not inhibited by any known endogenous serum factor, adaptive evolution of their hemostatic protein targets might be expected to have occurred in venom-resistant opossums.

**Figure 2:**
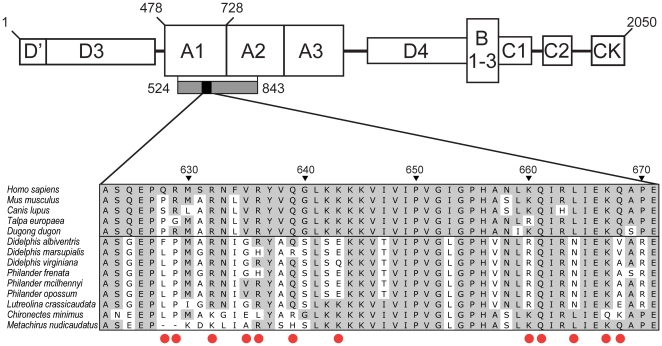
Structure of vWF showing botrocetin-binding sites. Schematic showing the structure of the mature vWF protein and its constituent domains (A, B, C, D, and CK; modified from [39]). Amino-acid residues are numbered 1–2050 corresponding to the human vWF sequence, with the A1 domain spanning residues 478–728. The region sequenced from opossums for this study includes part of the A1 and A2 domains and spans residues 524–843 (indicated with a grey box). The botrocetin-binding region (indicated with a black box) is located in the A1 domain and spans residues 623–671. Aligned amino-acid sequences of this region are shown for five placental taxa (*Homo*, *Mus*, *Canis*, *Talpa*, and *Dugong*) as well as members of the opossum tribe Didelphini (including species of *Didelphis*, *Philander*, *Lutreolina*, and *Chironectes*) and its sister taxon Metachirini (*Metachirus nudicaudatus*). Amino acids that are identical to vWF sequence from *Homo* are shaded in grey. The 12 amino-acid residues (positions 628, 629, 632, 635, 636, 639, 643, 660, 661, 664, 667, and 668) identified as critical for botrocetin binding in *Mus* [47] are indicated with red dots below the sequences.

Hemostasis is a complex process that involves formation of a platelet plug at the site of vascular injury (primary hemostasis) followed by clot formation and stabilization via the coagulatory cascade [38]. One of the key proteins in this sequence is von Willebrand Factor (vWF), a large multidomain glycoprotein that initiates platelet-plug formation by anchoring to exposed subendothelial collagen and then binding with the Ibα subunit of platelet glycoprotein Ib-IX-V [39]. Not surprisingly, several toxins isolated from hemorrhagic snake venoms target vWF, including both metalloproteinases and CLPs [37], [40], [41]. To date, the best-studied vWF-binding CLPs isolated from pitviper venom are botrocetin and aspercetin, both of which induce thrombocytopenia and contribute to systemic bleeding by binding with the A1 domain of vWF and enhancing its affinity for platelet glycoprotein Ibα [42]–[46]. Recently, three-dimensional models of the vWF-botrocetin complex have been developed, and specific residues in the A1 domain of mouse vWF that are crucial for botrocetin binding have been identified [47] (Fig. 2).

We used vWF sequences from a phylogenetic study of opossums [28] (Fig. 1a) to test for accelerated rates of adaptive evolution among opossums known to eat pitvipers and/or to be resistant to pitviper venom. To do so, we analyzed patterns of selection in a portion of the vWF gene comprising the A1 domain, including all of the sites that code for residues known to be necessary for botrocetin binding in humans and laboratory rodents. Our results support the notion that snake-venom components exert strong directional selection on the amino-acid sequences of targeted proteins in routinely envenomed species and lend credence to the commonly invoked but previously untested metaphor of a coevolutionary arms race. However, our results also suggest a more prominent defensive role for snake venom than is commonly acknowledged by most toxinologists.

## Results and Discussion

### Positive Selection on vWF in venom-resistant opossums

To test for adaptive evolution in opossum vWF sequences, we used codon-model-based branch-site tests of positive selection [48]. Given the phylogenetic distribution of venom resistance discussed above and shown in Fig. 1b, resistance presumably evolved somewhere along the branch separating *Metachirus* from the *Lutreolina* + *Didelphis* + *Philander* clade. However, the water opossum *Chironectes* has yet to be tested for venom resistance, so we have no prior knowledge that would allow us to unequivocally reconstruct the evolution of venom resistance along the branch leading to Didelphini (including *Chironectes*) or along the branch subtending the less-inclusive clade *Lutreolina* + *Didelphis* + *Philander*. For this reason, we performed two separate branch-site tests of selection: one that assigned all lineages of Didelphini to a class with the possibility of having positively selected sites (“foreground” branches [48]), and one that assigned all Didelphini except *Chironectes* to that class (see Methods). For both tests, all other branches in the didelphid phylogeny were treated as “background” branches that did not include a class of positively selected sites.

Based on these tests, we found strong evidence for positive selection on vWF in venom-resistant opossums. Specifically, a model that allows a proportion of sites to be under positive selection for Didelphini was a significantly better fit than the null model (no sites allowed to be under positive selection in any lineage), regardless of whether *Chironectes* was included among the venom-resistant lineages or not (test including *Chironectes*: 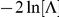 = 26.44, d.f. = 1, **p**≪0.01; test excluding *Chironectes*: 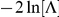 = 17.54, d.f. = 1, **p**≪0.01). Approximately 10% of sites in the analyzed fragment of vWF were estimated to be under positive selection in both scenarios (10.3% of sites have 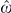 = 6.79 if *Chironectes* is included among the foreground lineages, whereas 9.6% have 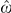 = 8.31 if *Chironectes* is excluded; Table 1). If *Chironectes* is included among the venom-resistant lineages, we identified nine sites that showed strong evidence (*P*≥0.95) of belonging to the class of positively selected sites, and an additional 16 sites with 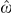
>1 using the less stringent criterion of 0.5<*P*<0.95 (Table 2). If *Chironectes* is excluded, five sites have 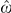
>1 with *P*≥0.95 (four of these were also identified when *Chironectes* was included among the foreground lineages).

**Table 1:** Results of branch-site tests for selection on vWF.

Model constraint	site class^1^	Proportion of sites	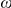 (background)	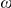 (foreground)
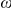 _2_ = 1	0	0.624 (0.610)	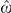 _0_ = 0.055 (0.060)	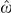 _0_ = 0.055 (0.060)
	1	0.176 (0.176)	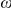 _1_ = 1	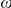 _1_ = 1
	2a	0.156 (0.166)	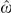 _0_ = 0.055 (0.060)	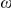 _2_ = 1
	2b	0.044 (0.048)	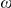 _1_ = 1	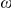 _2_ = 1
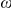 _2_>1	0	0.692 (0.700)	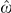 _0_ = 0.054 (0.060)	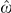 _0_ = 0.054 (0.060)
	1	0.205 (0.204)	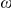 _1_ = 1	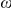 _1_ = 1
	2a	0.080 (0.074)	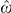 _0_ = 0.054 (0.060)	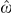 _2_ = 6.79 (8.31)
	2b	0.023 (0.022)	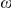 _1_ = 1	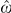 _2_ = 6.79 (8.31)

Parameter estimates for branch-site tests (H_0_: 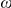
_2_ = 1; H_A_: 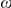
_2_>1) applied to vWF sequences with either the clade Didelphini (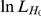 = −5436.01; 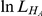 = −5422.79) or Didelphini excluding *Chironectes* (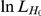 = −5440.51; 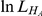 = −5431.74; parameter values in parentheses) designated as foreground lineages.

1 Site classes 0 and 1 comprise sites under purifying selection (0<
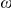
_0_<1) and neutral sites (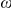
_1_ = 1), respectively in both foreground and background lineages. Site class 2 allows a proportion of positively selected sites in the foreground lineages, where 2a includes sites under purifying selection (0<
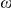
_0_<1) in the background lineages, 2b includes neutral sites in the background lineages. Both 2a and 2b allow a proportion of sites in the foreground lineages to be under positive selection (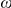
_2_>1).

**Table 2:** Results of Bayes-Empirical-Bayes analyses identifying positively selected sites.

Site^1^	Foreground branches exclude *Chironectes* 2	Foreground branches include *Chironectes* 2	Function^3^
Leu533	0.80	0.71	
Asp560	0.62	–	
Glu567	<0.50	0.75	
Gln590	**0.99**	**1.00**	
Thr601	<0.50	0.85	
Asp610	**1.00**	**0.99**	
Thr622	0.54	<0.50	
Gln625	0.65	<0.50	
Pro628	0.88	0.82	Botrocetin binding
Ala631	**0.95**	0.89	
Asn633	<0.50	**0.98**	
Val635	0.60	**0.98**	Botrocetin binding
Arg636	0.74	0.91	Botrocetin binding
Gln639	<0.50	0.73	Botrocetin binding
Lys642	0.71	0.50	
Lys643	**1.00**	**0.99**	Botrocetin binding
Ile647	0.84	0.69	
Ala657	0.57	<0.50	
Ser658	0.55	<0.50	
Leu664	0.69	0.57	Botrocetin binding
Gln668	0.89	**0.98**	Botrocetin binding
Ala669	0.84	0.77	
Pro670	0.79	0.70	
Ala674	**1.00**	**0.99**	
Ser692	0.93	**1.00**	
Leu694	<0.50	0.69	
Thr705	0.83	0.69	
Lys728	0.60	<0.50	
Lys753	<0.50	**0.95**	
Lys795	0.76	0.66	
Pro838	<0.50	0.69	

Sites in vWF that were identified as being under positive selection (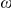
>1) with posterior probability >0.50 in Bayes-Emipircal-Bayes analyses with Didelphini (either excluding or including *Chironectes*) assigned as foreground lineages.

1 Numbered according to the mature vWF peptide in *Mus*.

2 Sites inferred to be under positive selection with *P*≥0.95 are shown in bold.

3 Positively selected sites corresponding to those involved in botrocetin binding in *Mus* are indicated. An additional five sites (Arg629, Arg632, Lys660, Gln661, Lys667) bind botrocetin in *Mus* but are not inferred to be positively selected in any of our analyses of opossum vWF sequences.

**Figure 3:**
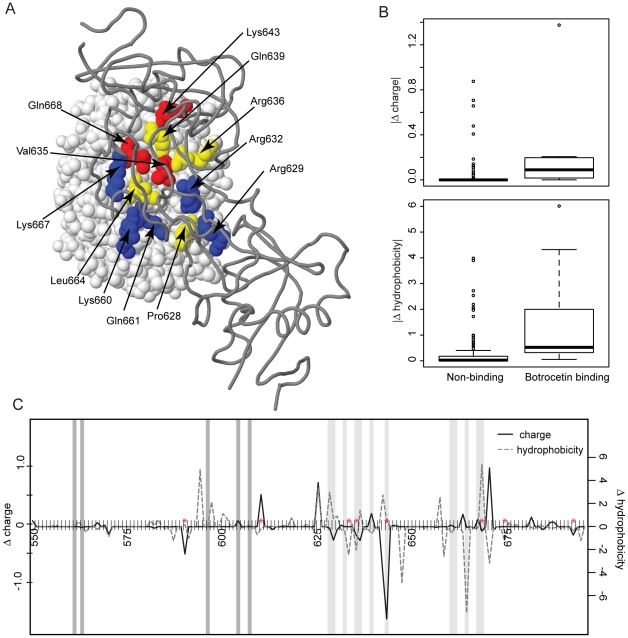
Structure and functional analyses of the vWF-A1 domain. A. The structure of the mouse vWF A1 domain complexed with botrocetin (Protein Data Bank file 1U0O [47]; image realized using Geneious v.5.0.3 [78]). The two chains of botrocetin are shown as a dark grey trace model. The vWF A1 domain is shown as a light grey spacefill model, with residues that are involved in botrocetin binding shown in color (yellow, red, or blue). Amino acid residues identified as being under positive selection in the lineage of venom-resistant opossums (Didelphini) are shown in red (*P*≥0.95) or yellow (0.5<*P*<0.95). Residues that are colored blue are involved in botrocetin binding but are not inferred to be under positive selection in opossums. **B**. Box plots of the absolute value of change in amino acid charge (top) and hydrophobicity (bottom) between venom-resistant and non-resistant taxa for sites of the vWF-A1 domain that bind botrocetin and those that do not. **C**. A site-by-site sliding window analysis along the vWF-A1 domain showing the average change in charge (solid line) and hydrophobicity (dashed line) between resistant and non-resistant taxa. Botrocetin-binding sites are indicated with pale grey bars, sites that bind platelet glycoprotein Ibα are dark grey, and sites that are under positive selection in venom-resistant opossums are indicated with red asterisks.

If CLPs are driving the rapid evolution of opossum vWF, then we might expect the particular sites that interact with botrocetin (or with homologous vWF-binding CLPs such as aspercetin [46], [49]) to show an elevated rate of evolution. Botrocetin engages vWF through two alpha helices (α5 and α6) on the exposed surface of the folded A1 domain [44]; in human and mouse models, site-directed mutagenesis studies have identified 12 sites distributed across these helices that are directly involved in botrocetin binding [47] (Fig. 3a). Sites that we identified as positively selected in venom-resistant opossums are disproportionately represented among these botrocetin-binding sites: of the 12 binding sites, three have high posterior probability (*P*≥0.95) of 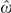
>1; of the remaining 193 sites in the A1 domain that do not bind botrocetin, four have high probability of being under positive selection. Thus, the proportion of botrocetin-binding sites showing evidence of positive selection in venom-resistant opossums is greater than would be expected by chance (G = 8.61, d.f. = 1; **p** = 0.01). However, if *Chironectes* is excluded from the lineage of venom-resistant species, then only one botrocetin-binding site is inferred as having a high probability of being under positive selection (G = 1.59; d.f. = 1; **p** = 0.29). Clearly, if *Chironectes* is venom resistant, the evidence from branch-site analyses for botrocetin (or other snake-venom CLPs with similar vWF-binding properties) as the causal selective agent is much stronger. Given the large number of amino-acid substitutions on the branch immediately ancestral to *Chironectes* in our phylogeny (Fig. 1b), and the fact that young pitvipers are often abundant along the rainforested stream margins frequented by water opossums (Voss, unpublished), it is plausible that this taxon has some degree of venom resistance, but external evidence (from *in vitro* or *in vivo* experimental challenges) will be necessary to resolve this issue.

Inferred physiochemical properties of amino-acid replacements in the A1 domain among venom-resistant opossums are also consistent with the hypothesis that selection has acted to inhibit binding with botrocetin or similar vWF-targeted CLPs. Existing models of the vWF-botrocetin complex implicate salt bridges, as well as water-mediated and ionic bonds in the interaction between the two molecules [44], [47]. Therefore, changes in residue charge should directly affect bond strength, and changes in amino-acid hydrophobicity could affect molecular conformation and steric interactions. To examine the possible functional significance of the observed changes in vWF, we quantified charge and hydrophobicity for each residue in the A1 domain for venom-resistant didelphines and non-resistant taxa (including other marsupials as well as placentals) and calculated the average change in these two properties between the two groups. For both charge and hydrophobicity, the magnitude of change between resistant and non-resistant taxa is significantly greater for the 12 sites that interact with botrocetin, as assessed by a Wilcoxon rank-sum test (Fig. 3b; charge: W = 216.5, **p** = 7.353e-08; hydrophobicity: W = 251.5, **p**<0.0001).

Several changes in the botrocetin-binding region of the A1 domain are unique to venom-resistant taxa and might play an important role in preventing botrocetin binding. For example, in both human and mouse models [44], [47], three tyrosine residues of botrocetin pack tightly against the A1 domain of vWF and appear to be critical for maintaining the binary complex: two of these tyrosines form ionic interactions with vWF residues Gln661 and Gln668; the third apposes site 628 (Pro in mouse; Arg in human) and Arg629. Of these sites, Gln661 shows little change in charge or hydrophobicity (Fig. 3c) and remains Gln in all resistant and most non-resistant taxa (Fig. 2). By contrast, site 668 (Gln in mouse and human) shows strong evidence of positive selection, and undergoes a dramatic change in average hydrophobicity between resistant and non-resistant taxa (Fig. 3c). This change is particularly notable for species of the venom-resistant genera *Didelphis* and *Philander*, where this site assumes strongly hydrophobic residues (Val or Ala; Fig. 2). Although neither site 628 nor 629 shows strong evidence of positive selection, both sites are, on average, more hydrophobic in venom-resistant didelphines than in non-resistant mammals. The remaining botrocetin-binding sites are predominantly positively charged in the human and mouse models and interact with negatively charged sites on botrocetin. Of these, site Lys643 undergoes the most dramatic change in average charge between resistant and non-resistant taxa, even though it is not identified as positively selected in the venom-resistant clade. Again, this change is most notable for species of *Didelphis* and *Philander* where, with one exception (a neutral Gln in *D. virginiana*), this position takes a negatively charged Glu residue (Fig. 2).

**Figure 4:**
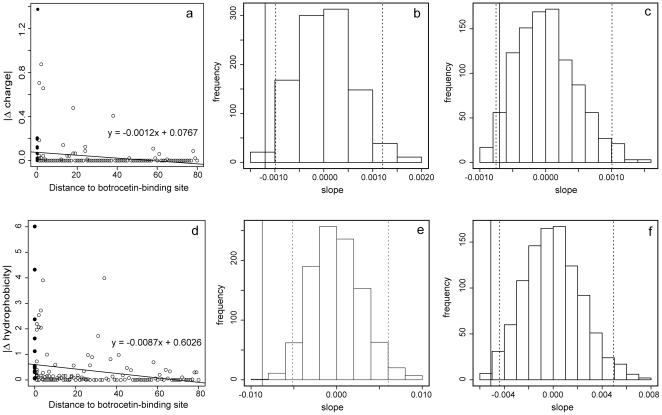
Change in amino-acid properties as a function of distance from botrocetin-binding sites. Plots of the absolute value of the average change in amino-acid charge (**a**) and hydrophobicity (**d**) between resistant and non-resistant taxa as a function of distance from a known botrocetin-binding site. Solid dots correspond to values at known botrocetin-binding sites; open circles indicate other sites in the A1 domain. For both physicochemical properties (charge, hydrophobicity), the magnitude of change is negatively correlated with distance from a known botrocetin-binding site. To test the significance of this correlation, we analyzed 1000 replicate datasets in which magnitude of change in each physicochemical property was randomized across the sequence. Histograms show the distribution of slope values for the best-fit regression lines through scatterplots of change in charge (**b**, **c**) or change in hydrophobicity (**e**, **f**) as a function of these randomly permuted distances. Permutations were performed with (**b**, **e**) and without (**c**, **f**) botrocetin-binding sites included. Dashed lines indicate the limits of the 95% confidence interval; solid lines correspond to the slope of the best-fit regression line based on the unpermuted data.

Depending on residue-specific substitution rates, adaptive evolution may or may not yield statistically compelling evidence for positive selection in branch-site tests. For example, certain changes in vWF occur only at the base of the clade comprising Didelphini, and others are single amino-acid changes within the clade; neither will necessarily be identified by phylogenetic tests of positive selection. Similarly, functionally important changes may not alter binding directly but through steric interactions caused by change at neighboring sites. A sliding-window analysis (Fig. 3c) suggests that changes in charge and hydrophobicity are clustered in the two regions corresponding to the botrocetin-interacting helices α5 and α6, even though many substitutions are at sites that do not directly bind botrocetin. In fact, the magnitude of change between resistant and non-resistant taxa is significantly negatively correlated with linear distance to known binding sites for both charge and hydrophobicity (Fig. 4). Even when binding sites are removed from the analysis, change in hydrophobicity is still significantly negatively correlated with distance to a known binding site (Fig. 4c, f). These results suggest that neighboring sites that may not interact directly with botrocetin through ionic interactions still experience changes in hydrophobicity that might affect the binding properties of the A1 domain, whereas sites that interact directly with botrocetin experience change in both charge and hydrophobicity. Interestingly, vWF sites that are involved in binding platelet glycoprotein Ibα are highly conserved between resistant and non-resistant taxa and experience little or no change in either of these amino-acid properties (Fig. 3c).

### Venom resistance as a genetically complex trait

Most previous explanations of snake-venom resistance in mammals have implicated endogenous venom-neutralizing factors isolated from blood serum or plasma; without known exceptions, these factors inactivate either snake-venom metalloproteinases or A_2_ phospholipases [31], [32], [34]. By contrast, no endogenous serum factors have yet been identified that inactivate snake-venom CLPs, the harmful physiological effects of which might be counteracted by other mechanisms in resistant species. Our results provide the first evidence for rapid adaptive evolution in any molecular target of snake-venom toxins, and they suggest that venom resistance is a more complex trait than is commonly recognized.

The only previous report of adaptive evolution in a venom-targeted molecule derives from sequence analyses of the nicotinic acetylcholine receptor (AChR) in snakes and in two snake-eating Old World mammals—mongooses (*Herpestes ichneumon*) and hedgehogs (*Erinaceus europaeus*)—that are resistant to α-bungarotoxin, a nonenzymatic neurotoxin found in cobra venom. These distantly related taxa all have nonaromatic amino-acid residues at two positions in the AChR protein that are crucial for α-bungarotoxin binding, whereas nonresistant species have aromatic residues at the same sites [50]. Although the experimental evidence for this adaptive interpretation is compelling [51], the published comparative data lack an appropriate phylogenetic context for statistical analysis, so it is not known whether the AChR locus has experienced sustained directional selection in venom-resistant clades. Interestingly, endogenous serum inhibitors of snake-venom metalloproteinases have also been discovered in mongooses and hedgehogs [34], suggesting that similarly complex mechanisms of venom resistance may have evolved convergently in opossums and in other mammals that eat snakes.

Because snake venoms typically contain dozens of toxic compounds [11], the evolution of venom resistance almost certainly requires adaptive changes at multiple loci. Both the genetic complexity of venom resistance and the need to maintain normal physiological functionality of venom-targeted molecules could constrain the evolution of immunity, although such evolution is perhaps to be expected in prey species routinely consumed by a locally abundant species of venomous snake (e.g., the California ground squirrel *Spermophilus beecheyi* [52])—or in predators for which venomous snakes are an important food source. The latter are of particular interest as a hitherto unrecognized factor in snake-venom evolution.

### A molecular arms race perhaps, but with whom?

Statistical evidence for adaptive evolution at the molecular level is rare [53], at least in part because successful detection of adaptation using comparative methods requires sites to be under strong and sustained positive selection in order to elevate *d_N_/d_S_* ratios above one [54]. To date, only a few classes of loci have been identified that appear to conform to this model of selection [55]; the best-known examples are genes involved in nonequilibrial coevolutionary contests, such as those between host and pathogen [56], [57] or between sperm and egg [58], [59]. In such cases, the analogy of an arms race is often invoked, perhaps appropriately where reciprocal adaptation and counter-adaptation have been convincingly demonstrated.

The arms-race metaphor might also be appropriate for explaining the rapid evolution of venom toxin genes if there were compelling evidence for rapidly evolving counter-adaptations (venom resistance) in routinely envenomed taxa. In fact, resistance to snake venom has been reported from a few prey species, mostly rodents [26], [52], [60], [61], but also from a wide array of animals that eat snakes [29], [62]–[65]. However, rapid adaptive evolution of genes conferring venom resistance has not previously been demonstrated, nor have the molecular substitutions responsible for venom resistance been examined in any substantive detail (except at the AChR locus described above).

Although our results are clearly consistent with an arms-race analogy for snake-venom evolution, the coevolutionary context in which this metaphor has previously been suggested explicitly assumes that venomous snakes are predators and that venom is a trophic adaptation [6]–[8], [11], [66]. However, this scenario is difficult to reconcile with theoretical work on the evolution of predator-prey interactions, which suggest that asymmetrical selection should result in more rapid evolution of attributes that contribute to prey survival than of attributes that increase predation success [12], [67], [68]. By contrast, other theoretical and empirical studies [69], [70] suggest that coevolutionary arms races are more likely to occur in predator-prey systems when prey are dangerous to predators. In such systems, selection may act to improve a predator's abilities to exploit dangerous prey, thereby establishing the basis for an arms race.

Several groups of snake-venom-resistant vertebrates—notably including various colubrid snakes (e.g., musaranas [*Clelia*] and kingsnakes [*Lampropeltis*]), opossums, mongooses, and hedgehogs—routinely prey on venomous snakes, and other species that have never been tested for venom resistance may frequently do so as well [71]–[72]). Therefore, it is plausible that snake venom has a significant defensive as well as a trophic role. If so, then the rapid evolution of snake-venom toxin genes should perhaps be reconsidered as the product of an arms race in which snakes are (at least sometimes) victims rather than exploiters [69].

## Materials and Methods

### vWF sequences and didelphid phylogeny

As part of a previous phylogenetic study [28], we sequenced a 963 bp region from exon 28 of the von Willebrand Factor gene from 41 species of didelphids (Genbank accession numbers FJ159328–FJ159370). For most of these taxa, the sequenced region begins with codon Met524 (numbered according to the *Mus* mature peptide sequence), which is 48 amino acids into the A1 domain of vWF (Fig. 2). For a few taxa (*Didelphis albiventris*, *D. marsupialis*, and seven other taxa that are not members of the tribe Didelphini), the sequenced region begins with codon Arg548. All of our didelphid sequences include complete sequences from the botrocetin-binding region (Fig. 2) and extend beyond the terminus of the A1 domain to Met843 in the neighboring A2 domain. The laboratory procedures we used for DNA amplification and sequencing this gene region are described elsewhere [28]. For phylogenetic analysis, vWF sequences were combined with DNA sequences from four additional protein-coding nuclear genes and with non-molecular characters, resulting in a combined-data matrix comprising 7320 bp plus 129 morphological characters. Mixed-model Bayesian analysis of this matrix [28] resulted in a well-resolved tree with high support values at most nodes (Fig. 1a). We used this topology as the basis for tests of positive selection described below.

### Selection tests

We used likelihood-based analyses of replacement and silent substitution rates [48] to test whether didelphid taxa known to eat pitvipers and/or to be venom resistant exhibited evidence of positive selection on vWF. Because we were interested in assessing whether or not vWF is under positive selection in a specific evolutionary lineage (members of the tribe Didelphini), we used a branch-site test specifically designed to test for episodic adaptive evolution [48], [73]. For this test, branches of the phylogeny are assigned *a priori* to either a “foreground” or a “background” class. For background lineages, codon sites are assigned to one of two classes: conserved (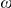
_0_, in which 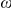 can assume values between 0 and 1) or neutral (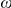
_1_, in which 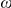 = 1). In the positive-selection model (model A [73], a proportion of sites for the foreground lineages can be assigned to an additional class of positively selected sites (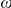
_2_, in which 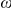
>1). This model is compared with a null model that disallows positively selected sites in the foreground lineages by fixing 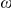
_2_ = 1 in those lineages.

We calculated the log-likelihood and parameter estimates for the null and alternative branch-site models using the *codeml* program of PAML ver. 4.4 [74]. The alternative model has four free parameters and the null model has three; because 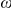
_2_ = 1 is fixed at the boundary of the parameter space of the alternative model, the relative fit of the two models (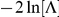) is assessed against a mixture of 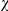 ^2^ distributions with 0 and 1 degrees of freedom (the 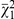 distribution [75]). However, as suggested [76], we used an unmodified 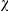 ^2^ distribution with one degree of freedom, which yields slightly more conservative critical values. Finally, we used a Bayes-Empirical-Bayes (BEB) method [73] to identify particular amino acid sites in the vWF protein that showed a high posterior probability of positively selected sites (those with 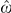
>1) in the foreground lineages.

### Comparative functional analyses

To examine the possible functional consequences of observed changes in vWF, we added sequences of the A1 domain from representative placental taxa available from Genbank (*Dugong* AAB51548; *Talpa* AAM82179; *Canis* NP0001002932; *Mus* CAB86200; *Marmota* CAB37847; *Lemur* CAC86209; *Hylobates* CAC86217; *Homo* NG009072) as well as other non-didelphid marsupials (*Sminthopsis crassicaudata* AY243412; *Murexia longicaudata* FJ159361; *Caenolestes fuliginosus* AY243403; *Rhyncholestes raphanurus* FJ159365; *Dromiciops gliroides* AY243407; *Echymipera kalubu* AY243405; *Perameles gunni* AY243411) to our existing matrix of didelphid sequences. For each sequence, we quantified per-site charge (1, 0 or −1) and hydrophobicity values [77]. For comparative purposes, we assigned each taxon to either a venom-resistant (*Chironectes*, *Lutreolina*, *Didephis*, and *Philander*) or non-resistant (all other taxa) class, and calculated the average change in each physicochemical property between the two.
